# Probiotics and Curcumin Did Not Alter Low‐Dose Streptozotocin‐Induced Hyperglycemia and Oxidative Stress in a Rat Model

**DOI:** 10.1002/fsn3.71624

**Published:** 2026-03-09

**Authors:** Cavdar Meliha, Yilmaz Muge, Ermiş Mustafa

**Affiliations:** ^1^ Department of Nutrition and Dietetics, Institute of Health Sciences Erciyes University Kayseri Turkey; ^2^ Department of Nutrition and Dietetics, Faculty of Health Sciences Erciyes University Kayseri Turkey; ^3^ Experimental Research Application and Research Center Erciyes University Kayseri Turkey

**Keywords:** curcumin, insulin resistance, oxidative stress, probiotics, type 2 diabetes mellitus

## Abstract

This study aimed to investigate the individual and combined effects of the multi‐strain probiotic VSL#3 and curcumin on glycemic control, insulin resistance, and oxidative stress (OS) in a rat model of T2DM induced by a high‐fat diet (HFD) and streptozotocin (STZ). Forty male Sprague–Dawley rats were divided into five groups: negative control, positive control (PC) induced with HFD and STZ, VSL#3 probiotic (PRO) (2.5 × 10^10^ CFU/day VSL#3), curcumin (CUR) (200 mg/kg/day curcumin), and combination of VSL#3 and curcumin (PRO+CUR) (2.5 × 10^10^ CFU/day VSL#3 + 200 mg/kg/day curcumin). At the end of 8 weeks, the study assessed the effects of these interventions on body weight, food intake, fasting blood glucose, and insulin resistance (HOMA‐IR). Additionally, serum and pancreatic tissue antioxidant parameters, were measured, including TAC, SOD, CAT, GP*x*, and MDA. VSL#3 and curcumin individually improved body weight, fasting blood glucose, and antioxidant enzyme activities. The PRO+CUR group showed the highest body weight gain and lower fasting blood glucose (353.83 ± 39.48 mg/dL) compared to the PC group (*p* < 0.05). However, the PRO+CUR combination did not yield the expected synergistic effects, likely due to curcumin's low bioavailability and prooxidant effects. Serum TAC was highest in the CUR group (0.78 ± 0.05 U/ng), whereas the PRO+CUR group showed reduced TAC (0.16 ± 0.02 U/ng). HOMA‐IR values increased in the PRO and CUR groups, but no significant change was observed in the PRO+CUR group. This study demonstrates the therapeutic potential of probiotics and curcumin in T2DM. Probiotics enhanced antioxidant defenses and reduced OS, but the combination with curcumin showed no synergistic effects, likely due to curcumin's bioavailability.

## Graphical abstract

In this study, a high‐fat diet/streptozotocin‐induced type 2 diabetes rat model was used to evaluate the individual and combined effects of the multi‐strain probiotic VSL#3 and curcumin on glycemic control, insulin resistance, and oxidative stress–related outcomes. Results indicated that VSL#3 and curcumin each modulated metabolic and antioxidant markers, whereas their combined administration did not provide a clear synergistic benefit, suggesting that curcumin‐related limitations (e.g., low bioavailability) may influence the overall response.

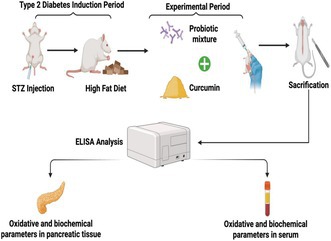

## 1 Introduction

Type 2 diabetes mellitus (T2DM) is a chronic metabolic disorder characterized by insulin resistance and impaired insulin signaling, which leads to elevated blood glucose levels and progressive β‐cell dysfunction. The global prevalence of T2DM is increasing alarmingly, creating substantial health and economic burdens on healthcare systems. Glycemic control represents a cornerstone in the management of T2DM, as persistent hyperglycemia is not merely a metabolic abnormality but a central driver of disease progression and diabetes‐related complications. Chronic elevation of blood glucose levels promotes excessive production of reactive oxygen species, accelerates the formation of advanced glycation end‐products, activates protein kinase C and polyol pathways, and induces chronic low‐grade inflammation. These interconnected mechanisms contribute to endothelial dysfunction, progressive β‐cell failure, and the development of microvascular and macrovascular complications, including retinopathy, nephropathy, neuropathy, and cardiovascular disease. Accordingly, effective glycemic control is essential not only for lowering blood glucose levels but also for mitigating oxidative stress (OS) and inflammatory processes that underlie T2DM pathophysiology (Brownlee 2001; Giacco and Brownlee 2010; Parmar et al. 2015).

Conventional pharmacological therapies, including oral hypoglycemic agents and insulin analogs, provide glycemic control but are often associated with long‐term side effects, patient compliance challenges, and significant costs (Davies et al. 2018; Khalid Khan and U Baig 2022). In addition to pharmacological approaches, a wide range of medicinal plants and complementary therapies have been explored for glycemic control in T2DM, primarily due to their antioxidant, anti‐inflammatory, and insulin‐sensitizing properties. However, recent comprehensive reviews indicate that the clinical application of many plant‐based and complementary interventions remains limited by heterogeneous study designs, lack of standardized formulations and dosing regimens, variable bioavailability, and insufficient long‐term safety and efficacy data. These limitations hinder their translation into evidence‐based clinical practice and highlight the need to focus on complementary strategies supported by more consistent mechanistic and clinical evidence, thereby providing a clear rationale for the selection of curcumin and probiotics as the therapeutic agents investigated in the present study (Mokgalaboni et al. 2023; Mokgalaboni and Phoswa 2023). Consequently, there is growing interest in safe, cost‐effective alternative strategies that address the underlying pathophysiology of T2DM, including OS and chronic inflammation, which are major contributors to disease progression (Blahova et al. 2021; Riddle et al. 2021).

Phytochemicals, nutraceuticals, and herbal products have gained significant popularity in recent years, primarily due to their minimal side effects and potential health benefits (Bulku et al. 2010). Recent studies highlight the potential of dietary interventions, functional foods, probiotics, and nutraceuticals with antioxidant properties as complementary therapies for T2DM management (Cicero et al. 2020; Mokgalaboni et al. 2024). Probiotics modulate gut microbiota, enhance intestinal barrier function, reduce systemic inflammation, and improve antioxidant capacity (Mattia and Merker 2008). Given the multifactorial nature of T2DM pathophysiology, combining interventions that act on distinct but interconnected pathways, such as gut microbiota modulation and OS reduction, may represent a promising therapeutic approach (DeFronzo et al. 2015). In this context, curcumin has emerged as a nutraceutical of particular interest due to its well‐documented antioxidant, anti‐inflammatory, and metabolic regulatory effects (Cicero et al. 2020; Hewlings and Kalman 2017). Importantly, the antihyperglycemic potential of curcumin has also been substantiated in clinical settings. A recent quantitative analysis of 18 randomized controlled trials involving 1382 patients with T2DM demonstrated that curcumin supplementation significantly reduced fasting blood glucose (mean difference: −11.48 mg/dL) and glycated hemoglobin (HbA1c; mean difference: −0.54%), accompanied by a significant reduction in C‐reactive protein levels compared with placebo, indicating concurrent improvements in glycemic control and systemic inflammation (Mokgalaboni et al. 2024). Taken together, these findings suggest that probiotics and curcumin may target overlapping yet distinct pathogenic mechanisms in T2DM, supporting the rationale for investigating their combined effects. Despite promising preclinical findings, the combined effects of probiotics and curcumin in T2DM models, particularly in high‐fat diet (HFD)‐induced insulin resistance, remain underexplored. The potential synergistic or antagonistic interactions and their effects on gut microbiota, short‐chain fatty acids (SCFAs), and antioxidant responses are not fully understood.

This study aimed to investigate the individual and combined effects of the multi‐strain probiotic VSL#3 and curcumin on glycemic control and OS in a rat model of HFD and STZ‐induced T2DM. The study evaluated the impact of these interventions on body weight, food intake, fasting blood glucose, and insulin resistance, assessed antioxidant status in serum and pancreatic tissue, and determined whether the combined use of probiotics and curcumin had synergistic, additive, or antagonistic effects on glycemic control and OS modulation. By addressing these objectives, this study fills a gap in the literature on the mechanistic interactions of probiotics and curcumin in T2DM.

## 2 Materials and Methods

### 2.1 Chemicals

Streptozotocin was used to induce T2DM, using the powdered product (Catalog No. 18‐883‐66‐4) obtained from Sigma‐Aldrich (St. Louis, MO, USA). To dissolve STZ, 1 mL of a 0.5 M, pH 6.0 buffer solution (Catalog No. J63950.AK) from Thermo Fisher Scientific (Waltham, MA, USA) was used. For orogastric gavage administrations, phosphate‐buffered saline (PBS) from Sigma‐Aldrich (St. Louis, MO, USA) with a pH of 7.4 (Catalog No. P4474) was used. The curcumin used in the study was a powdered substance obtained from Sigma‐Aldrich (St. Louis, MO, USA) (Catalog No. C1386).

### 2.2 Animals

A total of 40 male (8‐week‐old) Sprague–Dawley rats were obtained, maintained, and all experimental steps were carried out at Erciyes University Experimental Researches and Application Center (Kayseri, Turkey). Rats were housed in specific pathogen‐free rooms with an indoor temperature of 22°C ± 2°C, a relative humidity of 50% ± 10%, and a light cycle of 12 h light and 12 h dark. Rats in all groups were kept in single cages throughout the study period and fed ad libitum with feed and water.

The sample size was determined using a power analysis based on total antioxidant capacity (TAC) data measured in diabetic rats. Using G*Power v3.1.9.2, the required number of animals per group was calculated as 8 when the significance level (*α*) = 0.05 and the Type II error probability (*β*) = 0.10 (power = 1 − *β* = 0.90) were calculated (Pegah et al. 2021). The animals were randomly divided into five groups consisting of 8 rats each.

### 2.3 Diets and Treatment

Rats were fed two separate diets: a standard diet and a HFD. The HFD was produced by Arden Feed Industry Research and Experiment Trade Co. Ltd. (Ankara, Turkey) based on Research Diets' (DIO series) D12492 formulation; 60% of the total energy in this diet was derived from fat. The formulation of the chows used in the study is presented in Table 1. Curcumin was administered to rats in the CUR and PRO+CUR groups once daily for 28 days at a dose of 200 mg/kg dissolved in 1 mL of PBS solution via orogastric gavage. In addition, the probiotic mixture VSL#3 was administered to rats in the PRO and PRO+CUR groups via orogastric gavage once daily for 28 days at a dose of 2.5 × 10^10^ CFU dissolved in 1 mL of PBS solution. In order to induce stress similar to that caused by orogastric administration in the CUR, PRO, and PRO+CUR groups in the NC and PC groups, 1 mL of PBS solution was administered orogastrically once daily for 28 days to rats in these groups.

**TABLE 1:** Chow (diet) formulation.

Chow (diet) type	Standard chow (diet)		High fat chow (diet)	
%	g	kcal	g	kcal
Protein	20	20.3	26	20
Carbohydrate	64	63.9	26	20
Lipid	7	15.8	35	60
Total		100		100
kcal/g	4		5.24	
*Ingredients*				
Casein, 80 Mesh	—	—	200	800
Casein, 80 Mesh	200	800	—	—
l‐cystine	3	12	3	12
Corn starch	397	1590	—	—
Maltodextrin 10	132	528	125	500
Sucrose	100	400	68.8	275
Cellulose, BW 200	50	0	50	0
Soybean oil	70	630	25	225
T‐butyl hydroquinone	0.014	0	—	—
Lard	—	—	245	2205
Mineral mix	35	0	10	0
Dicalcium phosphate	—	—	1.3	0
Calcium carbonate	—	—	5.5	0
Potassium citrate	—	—	16.5	0
Vitamin mix	10	40	10	40
Choline bitartrate	2.5	0	2	0
FD&C Blue Dye#1	—	—	0.05	0
Total	1000	4000	773.85	4057

*Note:* All units in table are expressed in SI units. Grams (g) and kilocalories (kcal) are used consistently across all measurements. All values are reported as percentages of total weight or energy content.

To induce T2DM, rats in the PC, PRO, CUR, and PRO+CUR groups (excluding the NC group) were administered a single intraperitoneal (ip) injection of STZ dissolved in 1 mL citrate buffer at a dose of 30–35 mg/kg following a 4‐week HFD.

**FIGURE 1:**
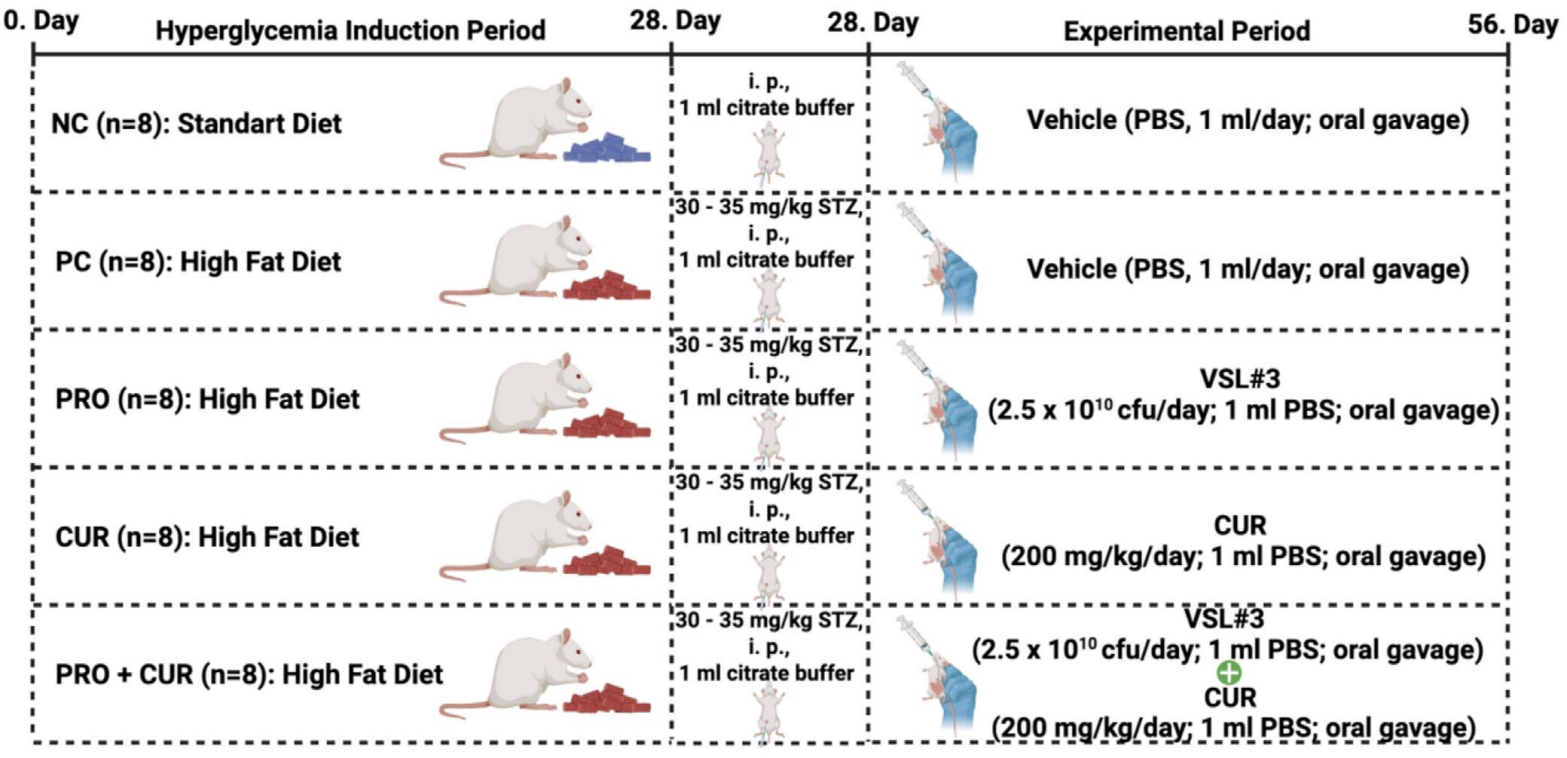
Flowchart of the study.

The groups included in the study and the protocols administered to each group are depicted in detail in the flow chart presented as Figure 1.

### 2.4 Preparation of Probiotic Extracts

In this study, VSL#3 Unflavored was utilized. Each sachet of VSL#3 Unflavored (4.4 g net) contains 450 billion probiotic bacteria, including *Streptococcus salivarius* subsp. *thermophilus* BT01, *Bifidobacterium breve* BB02, *Bifidobacterium animalis* subsp. *lactis* BL03, *Bifidobacterium animalis* subsp. *lactis* BL04, *Lactobacillus acidophilus* BA05, *Lactiplantibacillus plantarum* BP06, *Lacticaseibacillus paracasei* BP07, and *Lactobacillus helveticus* BD08, along with corn starch and silicon dioxide. The probiotic was stored at 2°C–8°C to maintain viability. Given that the anxiolytic and antidepressant‐like effects of *Bifidobacterium* and *Lactobacillus* species have been well documented in both rodents and humans, this specific probiotic formulation was selected for this study (Savignac et al. 2014). Previous research demonstrated that VSL#3, administered at a dose of 2.5 × 10^10^ CFU/day, successfully colonized the gut (Beilharz et al. 2018). Based on these findings, the same dosage was employed in this study. For administration, the probiotic was suspended in 1 mL of PBS per rat to achieve a final concentration of 2.5 × 10^10^ CFU/mL and delivered via orogastric gavage.

### 2.5 Streptozotocin‐Induced Diabetes Animal Model

Before the induction of diabetes with STZ, all experimental animals except Group 1 were fasted for 16 h, during which they had free access to drinking water. At the end of the fasting period, all animals were weighed and their actual body weights were recorded. To establish a T2DM model, STZ at a dose of 30–35 mg/kg dissolved in 1 mL of citrate buffer was administered as a single dose intraperitoneally (Zhang et al. 2017). Following the administration, rats were given water containing 10% glucose for the first 24 h to prevent hypoglycemia (Kurçer and Karaoğlu 2012). Fasting blood glucose levels were measured 72 h after STZ injection using blood samples taken from the tail vein of the animals with a GlucoLeader Enhance 2 glucometer (HDM Biomedical Inc., Taiwan) (Zahkok and Abo‐Elnaga 2016). Animals with fasting blood glucose levels of 250 mg/dL or higher were accepted as diabetic (hyperglycemic) (Abdelhafez and El‐Dahshan 2022).

### 2.6 Body Weight and Food Intake

Body weight and food intake were measured and recorded weekly for 8 weeks. Changes in body weight were calculated by minus the weight from the previous week from the actual body weight of each week. The total body weight gain was determined by summing the weight increases recorded each week from Week 0 to Week 8. Similarly, total food consumption was calculated by summing the amount of food consumed each week over the 8‐week period.

### 2.7 Fasting Blood Glucose Measurement and Insulin Resistance

Fasting blood glucose levels in rats were measured using a glucometer and blood glucose test strips, using blood samples taken from the tail vein on Days 28 and 56. Fasting blood insulin levels were determined on Day 56 using a blood sample taken from the tail vein and a commercial ELISA kit (BT‐LAB). Insulin resistance was calculated using the Homeostatic Model Assessment for Insulin Resistance (HOMA‐IR) method, using the following formula recommended by the Turkish Diabetes Foundation (2017):

$$\begin{array}{c} \text{HOMA}-\mathrm{IR}=\\ \;\frac{\text{Fasting blood glucose}\;\left(\mathrm{mg}/\mathrm{dL}\right)\times \text{Fasting blood insulin}\;\left(\mathrm{uIU}/\mathrm{mL}\right)}{405} \end{array}$$

In the assessment of insulin resistance, the threshold value for HOMA‐IR is accepted as 2.5; HOMA‐IR levels ≥ 2.5 are considered as the development of insulin resistance (Ermiş and Çiftci 2024).

### 2.8 Blood and Tissue Collection

On the last day of the 8‐week study period, rats were fasted for 12 h prior to killing. At the end of the fasting period, anesthetic agents were administered intraperitoneally. Ketamine (Ketasol, Richter Pharma AG, Austria) and xylazine (Rompun, Bayer, Germany) were administered at doses of 50 and 10 mg/kg, respectively, for anesthesia. After ensuring adequate anesthesia depth, a maximum cardiac blood sample was collected. The blood was then centrifuged at 3000 × *g* for 15 min at +4°C, and serum was obtained. Rats were killed by the cervical dislocation method, and the pancreas tissues were then quickly removed.

### 2.9 Analysis of Biochemical Parameters

Serum obtained from blood samples and the supernatant obtained by homogenizing pancreatic tissue were used to analyze various biochemical parameters. Serum TAC was analyzed using a commercial enzyme‐linked immunosorbent assay (ELISA) kit (Cat. No. MBS1600693) from Mybiosource (San Diego, USA). Superoxide dismutase (SOD, Cat. No. E0168Ra), catalase (CAT, Cat. No. E0869Ra), glutathione peroxidase (GP*x*, Cat. No. E1242Ra), glutathione reductase (GR, Cat. No. E1085Ra), malondialdehyde (MDA, Cat. No. E3442Ra), nuclear factor erythroid 2‐related factor 2 (NRF2, Cat. No. E1083Ra), Kelch‐like ECH‐associated protein 1 (KEAP1, Cat. No. E2124Ra), glucose (Cat. No. E2225Ra), and insulin (Cat. No. E0707Ra) levels were analyzed using commercial ELISA kits from BT‐LAB (Shanghai, China).

### 2.10 Statistical Analysis

The data obtained from the study were analyzed using SPSS (IBM Corp., Armonk, NY, USA) 25 software. All data were expressed as mean ± SD, and *p* value less than 0.05 was considered statistically significant. GraphPad Prism 10.2.3 (GraphPad Software, Massachusetts, USA) was used to convert the data into graphs. For normally distributed variables, one‐way analysis of variance (ANOVA) was used to compare more than two independent groups. In order to determine the group or groups that made a difference, Tukey's test was applied when the homogeneity of variance assumption was satisfied, and Tamhane's test was performed when the homogeneity assumption was not satisfied. For variables that did not show normal distribution, the Kruskal–Wallis test was used to compare more than two independent groups. The Bonferroni test was performed to determine the group or groups that made a difference. Effect sizes were reported for all tests: *η* ^2^ was used for ANOVA and *ε* ^2^ for Kruskal–Wallis. In addition to *p*‐values, 95% confidence intervals (95% CIs) for effect sizes and group means were provided. Table S1 present detailed including effect sizes, *p*‐values, and 95% CIs for each group comparison.

## 3 Results

### 3.1 Effects of the Treatments on Body Weight, Body Weight Gain, and Food Intake

**FIGURE 2:**
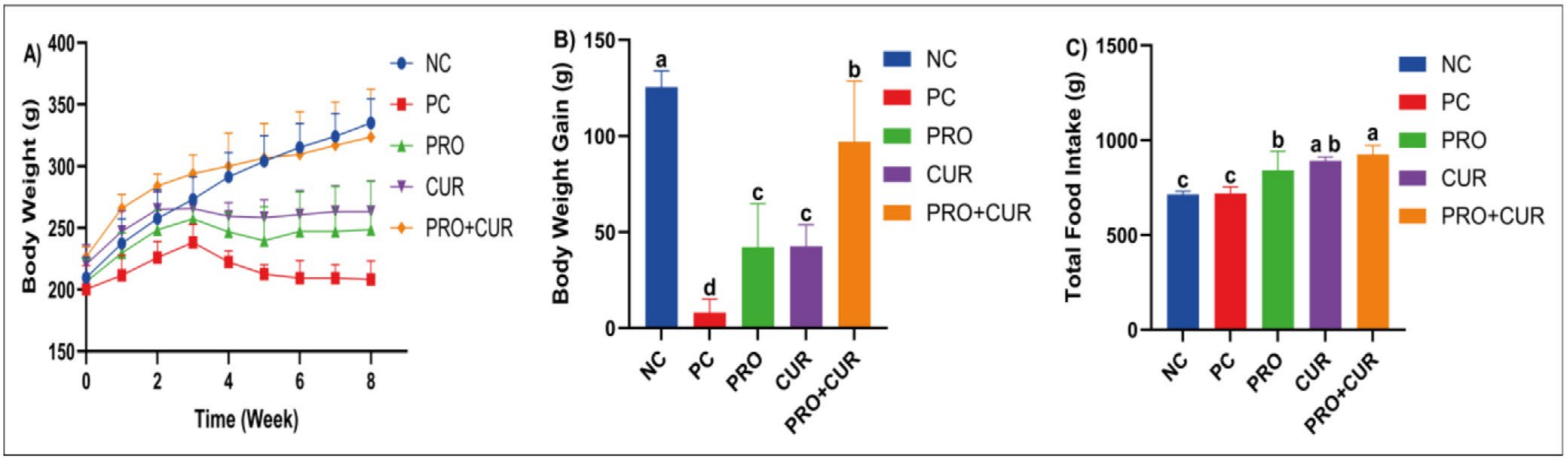
The effects of probiotics and curcumin on body weight, body weight gain, and total food intake. (A) Body weight during 8 weeks, (B) body weight gain during 8 weeks, (C) total food intake during 8 weeks. CUR, curcumin group; NC, negative control group; PC, positive control group; PRO, probiotic group; PRO+CUR, probiotic+curcumin group. Data are shown as mean ± SD (*n* = 8), whereas different letters signify substantially different values (*p* < 0.05).

As shown in Figure 2A, no significant difference in body weight was observed between groups during the first 4 weeks of hyperglycemia induction. However, after STZ administration, the NC group's mean body weight was significantly higher than that of the PC, PRO, and CUR groups in Weeks 5–8 (*p* < 0.001). Additionally, the PRO+CUR group's mean body weight was significantly higher than the PC, PRO, and CUR groups in Weeks 5–8 (*p* < 0.001). The NC group showed the highest weight gain (125.38 ± 8.55 g), whereas the PC group had the lowest (8.08 ± 6.98 g). The PRO (42.20 ± 22.61 g) and CUR (42.60 ± 11.18 g) groups had similar gains, which were higher than the PC group but lower than the PRO+CUR group (97 ± 31.57 g) (Figure 2B). The total food consumption of the NC (714.70 ± 17.17 g) and PC (719.32 ± 35.65 g) groups was lower than that of the other groups. The mean consumption of the PRO (841.80 ± 100.61 g) group was similar to that of the CUR (891.40 ± 20.11 g) group, but significantly lower than that of the PRO+CUR (925.31 ± 47.88 g) group (Figure 2C).

### 3.2 Effects of the Treatments on Blood Glucose Levels and Insulin Resistance

**FIGURE 3:**
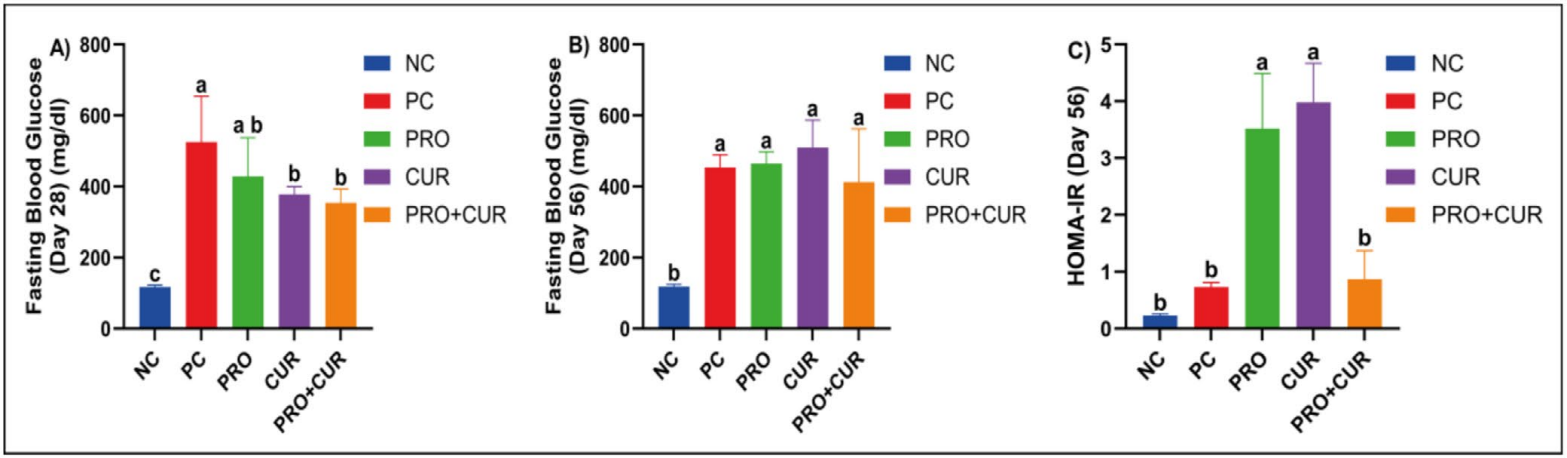
The effects of probiotic and curcumin on fasting blood glucose and HOMA‐IR levels. (A) Fasting blood glucose on Day 28, (B) fasting blood glucose on Day 56, (C) HOMA‐IR level on Day 56. CUR, curcumin group; PRO+CUR, probiotic+curcumin group; NC, negative control group; PC, positive control group; PRO, probiotic group. The comparison of parameters between groups was performed using one‐way ANOVA and different lowercase letters indicate statistically significant differences between groups (*p* < 0.05). Data are shown as mean ± SD (*n* = 8).

On Day 28, after hyperglycemia induction with high‐fat feed, fasting blood glucose levels of the PC (525.33 ± 129.32 mg/dL), PRO (428.00 ± 108.97), CUR (377.60 ± 21.78), and PRO+CUR (353.83 ± 39.48) groups were significantly higher than that of the NC (116.87 ± 5.54) group. The PC group's mean fasting blood glucose level was similar to that of the PRO group, but significantly higher than the means of the CUR and PRO+CUR groups. No significant difference was observed between the CUR and PRO+CUR groups (Figure 3A; Table S1). On Day 56, the mean fasting blood glucose levels of the PC (453.66 ± 35.27 mg/dL), PRO (464 ± 33.67), CUR (509.80 ± 77.36), and PRO+CUR (412 ± 150.13) groups were similar, but all were significantly higher than that of the NC (118 ± 7.23) group (Figure 3B; Table S1). On the last day, the HOMA‐IR score was higher in the PC (0.73 ± 0.08) and PRO+CUR (0.87 ± 0.5) groups than in the NC (0.23 ± 0.03) group, but this difference was not significant. However, the HOMA‐IR scores of the PRO (3.52 ± 0.97) and CUR (3.98 ± 0.69) groups were significantly higher than those of the NC, PC, and PRO+CUR groups (*p* < 0.05) (Figure 3C; Table S1).

### 3.3 Effects of the Treatments on Serum Biochemical Parameters

**FIGURE 4:**
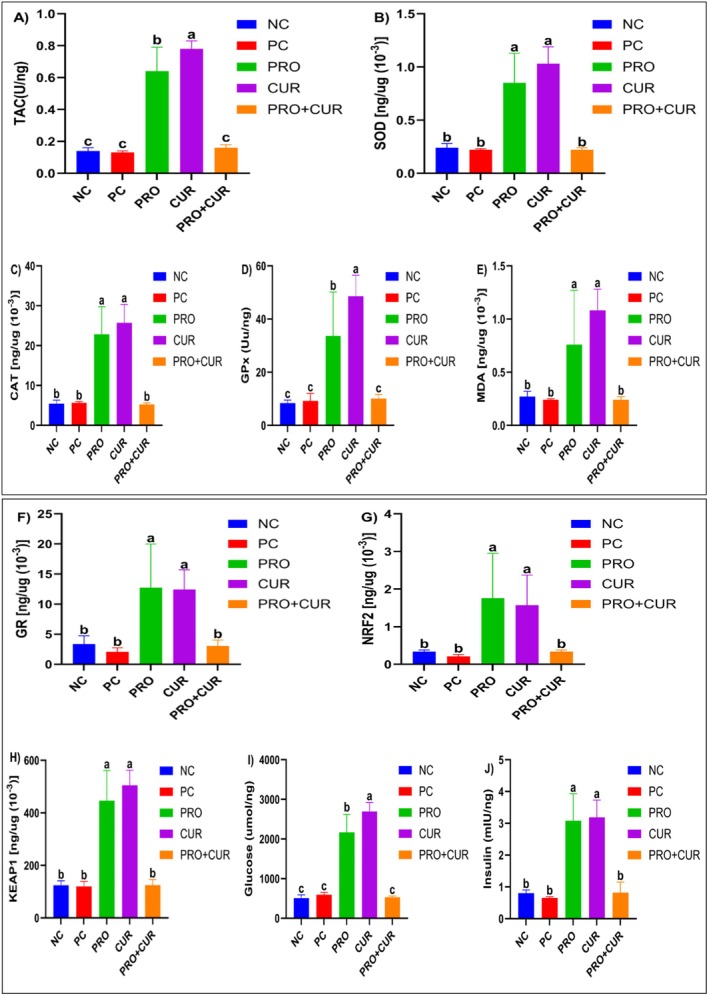
The effects of probiotic and curcumin on serum biochemical parameters. (A) Total antioxidant capacity (TAC); (B) superoxide dismutase (SOD); (C) catalase (CAT); (D) glutathione peroxidase (GP*x*); (E) malondialdehyde (MDA); (F) glutathione reductase (GR); (G) nuclear factor erythroid 2‐related factor 2 (NRF2); (H) Kelch‐like ECH‐associated protein 1 (KEAP1); (I) glucose; (J) insulin. CUR, curcumin group; NC, negative control group; PC, positive control group; PRO, probiotic group; PRO+CUR, probiotic+curcumin group. The comparison of parameters between groups was performed using one‐way ANOVA and Kruskal–Wallis tests. Different lowercase letters indicate statistically significant differences between groups (*p* < 0.05). Data are shown as mean ± SD (*n* = 8).

Figure 4A shows that the mean serum TAC level in the PRO+CUR group (0.16 ± 0.02 U/ng) was 23.1% and 14.3% higher than that of the PC (0.13 ± 0.01 U/ng) and NC (0.14 ± 0.02) groups, but these differences were not significant (Table S2). In contrast, the PRO group (0.64 ± 0.15) was significantly higher than the NC, PC, and PRO+CUR groups, but lower than the CUR (0.78 ± 0.05) group (*p* > 0.05). The mean serum SOD level in the PRO group (0.85 ± 0.28 [ng/μg × 10^−3^]) was significantly higher than that of the NC (0.24 ± 0.04), PC (0.22 ± 0.01), and PRO+CUR (0.22 ± 0.02) groups (*p* < 0.05). The CUR group (1.03 ± 0.16 [ng/μg × 10^−3^]) was 21.2% higher than PRO; however, this difference was not significant (Figure 4B; Table S2). The mean serum CAT level in the PRO (22.86 ± 6.91 [ng/μg × 10^−3^]) group was significantly higher than that in the NC (5.46 ± 0.85), PC (5.66 ± 0.28), and PRO+CUR (5.21 ± 0.41) groups (*p* < 0.05). The CUR group (25.73 ± 4.63 [ng/μg × 10^−3^]) was 12.6% higher than PRO, but this difference was not significant; similarly, there was no difference among NC, PC, and PRO+CUR (*p* > 0.05) levels (Figure 4C; Table S2).

The CUR group had the highest mean serum GP*x* level (48.52 ± 7.95 Uu/ng; *p* < 0.05). Although the PRO group (33.64 ± 16.53) had significantly lower GP*x* levels than the CUR group but higher than the NC (8.43 ± 1.07), PC (9.23 ± 2.81), and PRO+CUR (10.10 ± 1.52) groups, with no significant differences among these groups (Figure 4D; Table S2). Serum mean MDA levels were similar in the NC (0.27 ± 0.05), PC (0.24 ± 0.01), and PRO+CUR (0.24 ± 0.03) groups. The PRO group (0.76 ± 0.51) had significantly higher MDA levels than the others (*p* < 0.05), whereas the CUR group (1.08 ± 0.20) had higher levels than the PRO group, but not significantly (Figure 4E; Table S2). Serum mean GR levels in the PRO (12.77 ± 7.22 [ng/μg × 10^−3^]) and CUR (12.45 ± 3.25) groups were similar and significantly higher than those of the NC (3.36 ± 1.37), PC (2.08 ± 0.66), and PRO+CUR (3.05 ± 1.01) groups, with no significant differences among the latter (Figure 4F; Table S2). The PRO group had the highest serum NRF2 level (1.76 ± 1.19), significantly higher than the NC (0.34 ± 0.04), PC (0.21 ± 0.05), and PRO+CUR (0.34 ± 0.04) groups, but no difference between these groups. The CUR group (1.57 ± 0.80) had 10.8% lower NRF2 levels than the PRO group, but the difference was not significant, though CUR was still significantly higher than NC, PC, and PRO+CUR (*p* < 0.05) (Figure 4G; Table S2).

The mean serum KEAP1 levels in the PRO group (446.97 ± 114.05 [ng/μg × 10^−3^]) were 257.8%, 272.8%, and 256.8% higher, respectively, than those in the NC (124.91 ± 16.34), PC (119.91 ± 19.47), and PRO+CUR (125.28 ± 20.83) groups (*p* < 0.05). The CUR group (504.82 ± 58.09) had a 12.9% increase compared to PRO, but the difference was not significant. No significant differences were observed between the NC, PC, and PRO+CUR groups (Figure 4H; Table S2). Mean serum glucose levels were similar in the NC (507.50 ± 83.33), PC (594.96 ± 60.17), and PRO+CUR (531.98 ± 31.00) groups, but the PRO group (2166.90 ± 454.76) showed significantly higher values (*p* < 0.05). Although the CUR group (2694.54 ± 230.59) showed a 24.3% increase compared to PRO, this difference was not statistically significant (Figure 4I; Table S2). Mean serum insulin levels in the PRO (3.08 ± 0.85 mIU/ng) and CUR (3.19 ± 0.54) groups were similar (*p* > 0.05), but both were significantly higher than those in the NC (0.80 ± 0.10), PC (0.65 ± 0.04), and PRO+CUR (0.82 ± 0.33) groups (*p* < 0.05) (Figure 4J; Table S2).

### 3.4 Effects of the Treatments on Biochemical Parameters in Pancreatic Tissue

**FIGURE 5:**
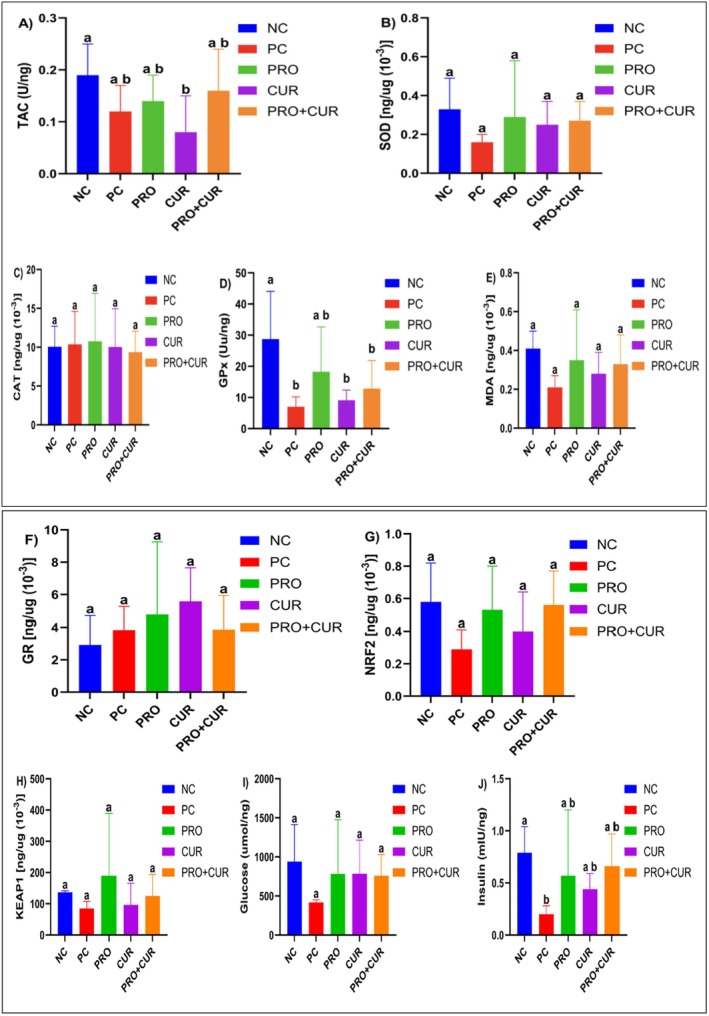
The effects of probiotics and curcumin on pancreatic tissue biochemical parameters. (A) Total antioxidant capacity (TAC); (B) superoxide dismutase (SOD); (C) catalase (CAT); (D) glutathione peroxidase (GP*x*); (E) malondialdehyde (MDA); (F) glutathione reductase (GR); (G) nuclear factor erythroid 2‐related factor 2 (NRF2); (H) Kelch‐like ECH‐associated protein 1 (KEAP1); (I) glucose; (J) insulin. CUR, curcumin group; NC, negative control group; PC, positive control group; PRO, probiotic group; PRO+CUR, probiotic+curcumin group. The comparison of parameters between groups was performed using one‐way ANOVA and Kruskal–Wallis tests. Different lowercase letters indicate statistically significant differences between groups (*p* < 0.05). Data are shown as mean ± SD (*n* = 8).

As shown in Figure 5A, the NC group (0.19 ± 0.06 U/ng) showed the highest and the CUR group (0.08 ± 0.07) the lowest TAC levels in pancreatic tissue, with a significant difference between them (Table S3). No significant differences were observed among the PC (0.12 ± 0.05), PRO (0.14 ± 0.05), and PRO+CUR (0.16 ± 0.08) groups, or between these and the NC or CUR groups. It was observed that the NC (0.33 ± 0.16 [ng/μg × 10^−3^]), PC (0.16 ± 0.04), PRO (0.29 ± 0.29), CUR (0.25 ± 0.12) and PRO+CUR (0.27 ± 0.10) groups had similar values when the mean SOD levels in pancreatic tissue were compared (*p* > 0.05) (Figure 5B; Table S3). The mean CAT levels were found to be similar among the NC (10.05 ± 2.66 [ng/μg × 10^−3^]), PC (10.36 ± 4.25), PRO (10.76 ± 6.18), CUR (10.02 ± 4.93), and PRO+CUR (9.36 ± 2.71) groups (*p* > 0.05) (Figure 5C; Table S3). Mean GP*x* levels were highest in the NC group (28.73 ± 15.33 U/ng), significantly higher than the PC (7.04 ± 3.15), CUR (9.11 ± 3.26), and PRO+CUR (12.84 ± 9.05) groups (*p* < 0.05). Although the GP*x* level in the PRO (18.21 ± 14.43) group was higher than that in the PC, CUR and PRO+CUR groups, and lower than that in the NC group, these differences were not statistically significant (Figure 5D; Table S3).

Similar to the SOD and CAT levels, the mean MDA levels showed no significant differences among the NC (0.41 ± 0.09 [ng/μg × 10^−3^]), PC (0.21 ± 0.06), PRO (0.35 ± 0.26), CUR (0.28 ± 0.11), and PRO+CUR (0.33 ± 0.15) groups (*p* > 0.05) (Figure 5E; Table S3). Additionally, the mean GR levels were also similar across the groups: NC (2.92 ± 1.79 [ng/μg × 10^−3^]), PC (3.84 ± 1.43), PRO (4.77 ± 4.49), CUR (5.58 ± 2.07), and PRO+CUR (3.86 ± 2.09) (*p* > 0.05) (Figure 5F; Table S3). The mean NRF2 levels were similar between the NC (0.58 ± 0.24 [ng/μg × 10^−3^]), PC (0.29 ± 0.12), PRO (0.53 ± 0.27), CUR (0.40 ± 0.24), and PRO+CUR (0.56 ± 0.21) groups (*p* > 0.05) (Figure 5G; Table S3). The highest KEAP1 level was in the PRO group (189.80 ± 199.51 [ng/μg × 10^−3^]); however, no significant differences were observed compared to the NC (136.93 ± 4.6), PC (84.59 ± 22.80), CUR (96.41 ± 69.12), and PRO+CUR (124.73 ± 69.58) groups (Figure 5H; Table S3). The mean glucose levels did not differ significantly between the groups: NC (939.69 ± 473.11 μmol/ng), PC (417.70 ± 32.81), PRO (783.03 ± 693.51), CUR (783.41 ± 431.28), and PRO+CUR (759.23 ± 272.06) (Figure 5I; Table S3). The mean insulin level was highest in the NC group (0.79 ± 0.25 mIU/ng) and significantly lower in the PC group (0.20 ± 0.08), with no significant differences between the PRO (0.57 ± 0.63), CUR (0.44 ± 0.15), and PRO+CUR (0.66 ± 0.31) groups (Figure 5J; Table S3).

## 4 Discussion

The present study investigated the individual and combined effects of the multi‐strain probiotic VSL#3 and curcumin on glycemic control, insulin resistance, and OS in a HFD/STZ‐induced rat model of T2DM. The results demonstrated that both VSL#3 and curcumin, when administered individually, improved fasting blood glucose levels and antioxidant enzyme activities. However, their combined administration did not produce synergistic metabolic benefits. Notably, the lack of synergism in the combined treatment may be attributed to curcumin's limited bioavailability. Additionally, curcumin's potential prooxidant effects could have influenced the results. These factors provide important context for interpreting the observed glycemic and antioxidant outcomes.

STZ induces toxicity in pancreatic β‐cells, suppressing insulin secretion, reducing glucose utilization, disrupting metabolism, and accelerating catabolic processes. These effects may lead to weight loss (Lenzen 2008). In this study, probiotics and curcumin partially alleviated STZ‐induced β‐cell damage. Additionally, both treatments increased pancreatic insulin levels. The PRO+CUR combination showed more significant improvements in insulin levels and body weight, suggesting a synergistic effect. Our findings are consistent with those reported by Hsieh et al. (2021). In their study, the administration of multi‐strain probiotics in STZ‐induced T2DM rats significantly increased pancreatic β‐cell size and serum insulin levels. However, no significant effect was observed on body weight (Hsieh et al. 2021). In another study by Na et al. (2011), the administration of 250 mg/kg curcumin for 7 weeks resulted in a 42.5% increase in insulin levels compared to the diabetic control group. Nevertheless, body weight was significantly decreased. The researchers associated this result with curcumin's ability to increase fatty acid oxidation. This process promotes energy utilization in skeletal muscles (Na et al. 2011).

In this study, the average body weight gain in the diabetes‐induced groups, which had higher daily average calorie intakes, was significantly lower compared to the control group fed a standard diet. This is thought to be due to impairments in insulin secretion. When glucose is not adequately taken up by cells, metabolic energy production decreases. As a result, body weight gain is adversely affected (Solis‐Herrera et al. 2021). In a study with a high single dose of STZ (50 mg/kg), rats treated with 0.6 mg/kg VSL#3 daily for 4 weeks showed no effect on body weight gain (Yesil et al. 2025). However, in this study, the same probiotic reversed the weight decrease caused by STZ. This difference may be due to variations in probiotic doses and diabetes models used. In another study with STZ‐induced diabetic rats, 100 mg/kg curcumin for 5 weeks had no significant effect on body weight gain compared to the diabetic control group. Unlike this study, that study did not use a HFD before STZ administration, suggesting different diabetes models. Additionally, the curcumin dose in that study was half the dose used in ours (Ermiş and Çiftci 2024).

Studies have shown that HFD and STZ‐induced diabetes models increase OS levels. These models also significantly decrease antioxidant enzyme activities and TAC in tissues and serum (Yan et al. 2020; Lee et al. 2021). In this study, similar declines were observed in the PC group, which was administered STZ without any intervention. Experimental studies in which T2DM was induced using a HFD and STZ have shown dysbiosis in the gut microbiota of diabetic animals. There were significant reductions in the relative abundance and diversity of bacterial genera such as *Lactobacillus* and *Bifidobacterium*. These genera play an important role in alleviating OS and improving glycemic control (Qumsani 2025). Yin et al. (2020) found that in diabetic mice, HFD and STZ administration reduced the relative abundance of Bacteroidetes in the gut microbiota. At the same time, there was an increase in the Proteobacteria phylum. The increase in the relative abundance of Proteobacteria leads to an increase in the synthesis of lipopolysaccharide molecules, which are endotoxin‐like compounds (Yin et al. 2020). The increase in lipopolysaccharide levels, characteristic of endotoxemia, reduces the expression of tight junction proteins. These proteins, such as occludin, zonula occludens‐1, and claudin‐1, are structural elements of the intestinal barrier. As a result, the intestinal barrier is disrupted, leading to leaky gut syndrome (Ma et al. 2021). In this syndrome, increased intestinal permeability results in the release of pro‐inflammatory cytokines such as IL‐1β, TNF‐α, and IL‐6 from the colon. These cytokines then enter the systemic circulation and trigger inflammatory responses. This condition increases systemic OS, which negatively affects the activity of antioxidant enzymes and TAC in the serum (Aleman et al. 2023). In this study, although the effects of the VSL#3 product on the taxonomic elements of the microbiota and inflammatory parameters were not determined, serum antioxidant enzyme levels and TAC values were significantly increased in the group treated with the probiotic alone.

Probiotics enhance intestinal barrier function by increasing tight junction protein expression, modulating gut microbiota, and reducing intestinal permeability. This prevents endotoxin passage and inhibits pro‐inflammatory cytokine production, thus reducing OS (Casula et al. 2023). In rats with acute intestinal damage, VSL#3 treatment reduced plasma TNF‐α and endotoxin levels. It also increased the expression of ZO‐1 and occludin in the small intestine, strengthening the barrier (Chang et al. 2013). Another possible mechanism by which probiotic strains or mixtures increase antioxidant enzyme activity and TAC levels is by enhancing the production of SCFAs. SCFAs activate regulatory T cells (Tregs), which suppress inflammatory responses and strengthen antioxidant defense by increasing the production of anti‐inflammatory cytokines such as IL‐10 (Wang et al. 2021). A study in dysbiosis‐induced mice showed that VSL#3 significantly increased the levels of Tregs and the anti‐inflammatory cytokine IL‐10 in the small intestine and colon (Ekmekciu et al. 2017). Probiotics are also known to stimulate glutathione production by increasing SCFA levels in the colon. Elevated glutathione levels play a significant role in neutralizing free radicals and increasing antioxidant enzyme levels, such as GR and GP*x* (Lutgendorff et al. 2008). In a study with obesity‐induced mice on a HFD, an increase in the relative abundance of butyrate‐producing bacteria in the gut microbiota and a significant rise in plasma butyrate levels were observed following VSL#3 administration (Yadav et al. 2013). These mechanisms support the potential of probiotics to enhance antioxidant capacity and reduce systemic OS.

In this study, VSL#3 significantly affected serum TAC, SOD, CAT, GP*x*, and GR levels. However, no effect was observed in pancreatic tissue. This suggests that VSL#3 failed to trigger the metabolic mechanisms needed to increase antioxidant capacity and reduce free radical damage. As a result, it was ineffective in reducing pancreatic OS. One key indicator of increased OS in the pancreas due to HFD and STZ is insulin resistance. Although serum and pancreatic insulin levels were elevated in the PRO group compared to the diabetic control group, this did not reduce serum glucose levels. This is linked to insulin resistance in the PRO group, as indicated by high HOMA‐IR values. The elevated insulin levels were ineffective at lowering glucose.

In vivo studies have shown that VSL#3 can reduce HOMA‐IR and serum glucose levels, or have no effect (Chong et al. 2021; Pegah et al. 2021). However, in this study, VSL#3 increased serum glucose and HOMA‐IR levels. This discrepancy is thought to be related to increased production of SCFAs, particularly acetate, in the colon following VSL#3 administration. Excessive acetate production from increased acetate‐fermenting bacteria may contribute to insulin resistance by enhancing gluconeogenesis in the liver (Moffett et al. 2020). In a dysbiosis‐induced knockout mouse study, it was shown that a dose of 2.25 × 10^9^ cfu/day of VSL#3 reduced colonic inflammation and strengthened intestinal barrier function. Additionally, it was reported that VSL#3 increased the abundance of acetate‐producing bacteria in the gut microbiota and elevated acetate levels in the colon. This was stated to suppress the production of pro‐inflammatory cytokines and chemokine responses (Kumar et al. 2017). In this study, the VSL#3 dose (2.5 × 10^10^ CFU/day) was 11 times higher than that used in the other study. Although SCFA analysis was not performed, it is suggested that the unusually high levels of acetate may be related to the inconsistent results in serum glucose and HOMA‐IR.

Probiotic strains, either alone or in mixtures, generally reduce pro‐inflammatory parameters and increase anti‐inflammatory ones in serum. However, the literature reports that probiotics can increase pro‐inflammatory cytokines. Contrary to expectations, they may also exhibit prooxidant effects, depending on the strain type, dose, and duration (Rocha‐Ramírez et al. 2017). An experimental study with *Lactobacillus reuteri* ATCC 6475 showed that both the live form and its surface protein compound increased pro‐inflammatory components like IL‐8, TNF‐α, and NF‐κB in monocyte cell lines (Jensen et al. 2015). In this study, VSL#3 increased serum MDA levels in the PRO group. This is thought to be due to the high dose and specific properties of the probiotic strains, contrary to literature findings.

In this study, curcumin alone increased TAC and enzyme activities in serum, but not in pancreatic tissue, likely due to its low bioavailability. Curcumin is a hydrophobic, lipophilic compound with minimal solubility in water at room temperature, particularly under acidic and neutral pH conditions. Its water solubility has been reported to be approximately 11 ng/mL. In order to achieve optimal bioavailability and therapeutic efficacy, the solubility of curcumin must be increased through metabolic processes (Stohs et al. 2020). Curcumin undergoes phase II metabolic reactions like glucuronidation and sulfation, rapidly metabolized in the liver and kidneys, and is eliminated before reaching target tissues. Additionally, curcumin's unstable structure reduces its circulating levels and duration. These factors limit its effectiveness (Stohs et al. 2019; Chen et al. 2020). To enhance curcumin's bioavailability, studies have explored innovative formulations such as liposomes, nanoparticles, and nanoemulsions, which improve solubility, extend circulation time, and boost effectiveness in target tissues (Hafez Ghoran et al. 2022). However, in this study, curcumin was administered in PBS without such enhancements. This limitation reduced its absorption in the intestines and hindered its ability to reach target tissues like the pancreas. Despite entering circulation and affecting serum parameters, curcumin's bioavailability was restricted.

In this study, curcumin administration increased serum MDA and worsened glucose, insulin, and HOMA‐IR values, contrary to literature findings. This may be due to curcumin's low bioavailability and high dosage. In experimental cancer models, high‐dose, long‐term curcumin administration has been shown to increase ROS levels. It also exhibits prooxidant effects, and induces metabolic toxicity, triggering apoptosis (Wolnicka‐Glubisz and Wisniewska‐Becker 2023; Hu et al. 2023). In T2DM models induced by HFD and low‐dose STZ, curcumin doses of 6–50 mg/kg/day were used, along with bioavailability‐enhancing nanoformulations (Abdulmalek et al. 2021; Chen et al. 2022; Li et al. 2024). In contrast, this study used a high dose of 200 mg/kg/day curcumin without any bioavailability‐enhancing solvent. It is believed that, due to its low absorption, curcumin, although present in high amounts in the colon, increased OS levels. This led to impairments in HOMA‐IR, serum glucose, insulin, and MDA levels.

To the best of our knowledge, no previous studies have investigated the effects of combined probiotic and curcumin administration on glycemic control and antioxidant parameters in a model of T2DM induced by a HFD and low‐dose STZ. In this study, curcumin and VSL#3 individually improved all serum parameters compared to the diabetic control group. However, in the PRO+CUR group, the metabolic effects observed when each intervention was administered alone were significantly reduced. This may be due to the interaction between probiotics, which modulate endotoxin passage and increase anti‐inflammatory cytokines and SCFAs (such as acetate). Curcumin's low bioavailability and prooxidant effects at high doses may also have contributed, attenuating the antioxidant benefits.

NRF2 is a transcription factor crucial for regulating antioxidant enzymes and protecting against OS by inducing antioxidant response elements (Uruno et al. 2013). Under OS conditions, KEAP1 suppresses NRF2. However, under increased OS, KEAP1's cysteine residues are modified, releasing the inhibition on NRF2 (Moon et al. 2020). In rats with renal ischemia–reperfusion injury, VSL#3 modulated tight junction proteins, strengthened the intestinal barrier, preserved antioxidant levels, and downregulated NRF2 and KEAP1 expression (Ding et al. 2019). In T2DM rats, curcumin increased serum SOD and GP*x* activity, upregulated NRF2, and downregulated KEAP1 in cardiac tissues (Wu et al. 2022). In this study, serum TAC and antioxidant enzyme levels increased in the PRO and CUR groups, with NRF2 levels significantly elevated. However, no similar effect was observed in pancreatic tissue following STZ administration. In the PRO and CUR groups, KEAP1 levels increased in parallel with NRF2, which is contrary to previous findings in the literature. In the PRO+CUR group, the expected effects were absent, and NRF2 and KEAP1 levels were decreased. This may be due to curcumin's low bioavailability and its high‐dose prooxidant effects, in combination with the high‐dose probiotics.

Despite the rigorous design, several limitations should be acknowledged. Although antioxidant status was assessed in serum and pancreatic tissue, other metabolically relevant organs, like liver and intestine, were not analyzed. This limits the understanding of systemic effects. Curcumin was administered in PBS without bioavailability‐enhancing strategies. This may have restricted its tissue distribution, particularly in the pancreas, and contributed to its limited efficacy at the target site. Gut microbiota profiling was not performed, which limits the mechanistic understanding of probiotic effects and their interaction with curcumin. Additionally, the use of a HFD combined with a single high‐dose STZ injection may not fully replicate the progressive β‐cell dysfunction characteristic of human T2DM. Finally, the preclinical nature and relatively short duration of the interventions limit the direct extrapolation of the findings to humans.

## 5 Conclusion

In conclusion, this study suggests that VSL#3 and curcumin, when administered individually, may improve glycemic control and antioxidant capacity in a HFD and STZ‐induced T2DM rat model. However, their combination reduced some benefits, possibly due to curcumin's low bioavailability, its high‐dose prooxidant effects, and probiotic‐induced metabolic modulation. The lack of synergy may stem from the low bioavailability of curcumin, which is a limitation of the current study. Future strategies to increase curcumin bioavailability, such as the use of bioavailability enhancers like piperine, may enhance the efficacy of this combination. Given the low bioavailability of the powdered curcumin used in this study, carrier systems such as nanoemulsions or nanoparticles should be investigated to increase the circulation time of curcumin. This will improve its ability to reach target tissues and organs, thereby improving its therapeutic potential. Probiotics likely enhance antioxidant defense and glycemic regulation through gut microbiota modulation, T‐cell activation, and increased anti‐inflammatory cytokines. Curcumin's limited bioavailability likely restricted its effects, particularly in the pancreas. This study provides insights into the therapeutic potential of probiotics and curcumin for T2DM, emphasizing the need for optimized dosing and delivery methods. These findings provide valuable insights into the potential of probiotics and curcumin as complementary therapeutic agents and may inform dosing strategies and protocols for future preclinical and clinical studies aimed at improving T2DM management. Future research should focus on long‐term interventions, advanced curcumin delivery systems, gut microbiota profiling, and human studies. Overall, these findings suggest that integrating probiotics and curcumin could enhance T2DM management by reducing OS and improving glycemic control.

## Author Contributions

Conceptualization: C.M. and Y.M. Data curation: C.M. Formal analysis: C.M. and E.M. Investigation: C.M. Methodology: C.M., Y.M., and E.M. Software: C.M. Supervision: Y.M. and E.M. Visualization: C.M. Writing – original draft: C.M., Y.M., and E.M. Writing – review and editing: C.M., Y.M., and E.M.

## Funding

This work was financially supported by the Erciyes University Scientific Research Fund (ERU‐BAP, Project Number: TDK‐2023‐13279).

## Ethics Statement

The necessary scientific permission for the study was granted by Erciyes University Animal Experiments Local Ethics Committee (Kayseri, Turkey) with ethical approval dated 06.09.2023 and decision number 23/176. In the care, feeding, and all other experimental processes of the animals in the study, the animal experiment directives of the European Union numbered 2010/63/EU were applied.

## Conflicts of Interest

The authors declare no conflicts of interest.

## Supporting information


**Table S1:** The effects of probiotic and curcumin on fasting blood glucose and HOMA‐IR levels.
**Table S2:**. The effects of probiotic and curcumin on serum biochemical parameters.
**Table S3:**. The effects of probiotic and curcumin on pancreatic tissue biochemical parameters.

## Data Availability

The data that support the findings of this study are available from the corresponding author upon reasonable request.
