# Correction to “Probiotics and Curcumin Did Not Alter Low‐Dose Streptozotocin‐Induced Hyperglycemia and Oxidative Stress in a Rat Model”

**DOI:** 10.1002/fsn3.71964

**Published:** 2026-05-27

Cavdar, M., M. Yilmaz, and M. Ermiş. 2026. “Probiotics and Curcumin Did Not Alter Low‐Dose Streptozotocin‐Induced Hyperglycemia and Oxidative Stress in a Rat Model.” *Food Science & Nutrition* 14, no. 3: e71624. https://doi.org/10.1002/fsn3.71624.

The first name and family name of the authors are corrected to “Meliha Cavdar”, “Muge Yilmaz”, and “Mustafa Ermiş”.

The online version of this article has been corrected accordingly.

We apologise for this error.

