# Supplementary material for: Probiotics and Curcumin Did Not Alter Low‐Dose Streptozotocin‐Induced Hyperglycemia and Oxidative Stress in a Rat Model
Source: Food Sci Nutr. 2026 Mar 9;14(3):e71624. doi: 10.1002/fsn3.71624 (PMC12971617; doi:10.1002/fsn3.71624)
Supplement: Supplementary file 1 — Table S1: The effects of probiotic and curcumin on fasting blood glucose and HOMA‐IR levels. Table S2:. The effects of probiotic and curcumin on serum biochemical parameters. Table S3:. The effects of probiotic and curcumin on pancreatic tissue biochemical parameters. [file FSN3-14-e71624-s001.docx]

**Table S1.** The effects of probiotic and curcumin on fasting blood glucose and HOMA-IR levels

| **Biochemical Parameters** | **^1^NC (n = 8)** | **^2^PC (n = 8)** | **^3^PRO (n = 8)** | **^4^CUR (n = 8)** | **^5^PRO+CUR (n = 8)** | **Test *p***  **value** | **Effect**  **Size** |
| --- | --- | --- | --- | --- | --- | --- | --- |
|  | **mean ± s.d. (CI)** | **mean ± s.d. (CI)** | **mean ± s.d. (CI)** | **mean ± s.d. (CI)** | **mean ± s.d. (CI)** |  |  |
| **FBG – Day 28 (mg/dl)** | 99.25 ± 7.16^c^  (95%CI: 93.25-105.24) | 525.33 ± 129.32^a^  (95%CI: 204.06-846.59) | 428.00 ± 108.97^ab^  (95%CI: 292.69-563.30) | 377.60 ± 21.78^b^  (95%CI: 350.54-404.65) | 353.83 ± 39.48^b^  (95%CI: 312.39-395.27) | <0.001 (F) | 0.868 (η^2^) |
| **FBG – Day 56 (mg/dl)** | 118.00 ± 7.23^b^  (95%CI: 111.95-124.04) | 453.66 ± 35.27^a^  (95%CI: 366.03-541.29) | 464.00 ± 33.67^a^  (95%CI: 422.18-505.81) | 509.80 ± 77.36^a^  (95%CI: 413.74-605.85) | 412.00 ± 150.13^a^  (95%CI: 254.43-569.56) | <0.001 (F) | 0.825 (η^2^) |
| **HOMA-IR – Day 56** | 0.23 ± 0.03^b^  (95%CI: 0.2042-0.2669) | 0.73 ± 0.08^b^  (95%CI: 0.5183-0.9494) | 3.52 ± 0.97^a^  (95%CI: 2.3203-4.7365) | 3.98 ± 0.69^a^  (95%CI: 3.1299-4.8490) | 0.87 ± 0.50^b^  (95%CI: 0.3158-1.4417) | <0.001 (F) | 0.903 (η^2^) |

Fasting Blood Glucose (FBG); Insulin Resistance (HOMA-IR); (1) NC, Negative Control group; (2) PC, Positive Control group; (3) PRO, Probiotic group; (4) CUR, Curcumin group; (5) PRO+CUR, Probiotic+Curcumin group; CI, Confidence interval; F, One-way ANOVA analysis; s.d., standard deviation. Data are shown as mean ± standard deviation (n = 8), whereas different letters signify substantially different values (*p*<0.05).

**Table S2.** The effects of probiotic and curcumin on serum biochemical parameters

| **Biochemical Parameters** | **^1^NC (n = 8)** | **^2^PC (n = 8)** | **^3^PRO (n = 8)** | **^4^CUR (n = 8)** | **^5^PRO+CUR (n = 8)** | **Test *p***  **value** | **Effect**  **Size** |
| --- | --- | --- | --- | --- | --- | --- | --- |
|  | **mean ± s.d. (CI)** | **mean ± s.d. (CI)** | **mean ± s.d. (CI)** | **mean ± s.d. (CI)** | **mean ± s.d. (CI)** |  |  |
| **TAC (U/ng)** | 0.14 ± 0.02^c^  (95%CI: 0.1217-0.1625) | 0.13 ± 0.01^c^  (95%CI: 0.1189-0.1410) | 0.64 ± 0.15^b^  (95%CI: 0.5179-0.7651) | 0.78 ± 0.05^a^  (95%CI: 0.7425-0.8213) | 0.16 ± 0.02^c^  (95%CI: 0.1455-0.1822) | <0.001 (F) | 0.947 (η^2^) |
| **SOD [ng/ug(*10^-3^)]** | 0.24 ± 0.04^b^  (95%CI: 0.2091-0.2803) | 0.22 ± 0.01^b^  (95%CI: 0.2090-0.2349) | 0.85 ± 0.28^a^  (95%CI: 0.6154-1.0852) | 1.03 ± 0.16^a^  (95%CI: 0.8973-1.1632) | 0.22 ± 0.02^b^  (95%CI: 0.2059-0.2469) | <0.001 (F) | 0.869 (η^2^) |
| **CAT [ng/ug(*10^-3^)]** | 5.46 ± 0.85^b^  (95%CI: 4.7490-6.1846) | 5.66 ± 0.28^b^  (95%CI: 5.4243-5.8980) | 22.86 ± 6.91^a^  (95%CI: 17.064-28.646) | 25.73 ± 4.63^a^  (95%CI: 21.857-29.606) | 5.21 ± 0.41^b^  (95%CI: 4.8613-5.5577) | <0.001 (F) | 0.875 (η^2^) |
| **GPx (Uu/ng)** | 8.43 ± 1.07^c^  (95%CI: 7.5299-9.3339) | 9.23 ± 2.81^c^  (95%CI: 6.8772-11.581) | 33.64 ± 16.53^b^  (95%CI: 19.797-47.465) | 48.52 ± 7.95^a^  (95%CI: 41.874-55.164) | 10.10 ± 1.52^c^  (95%CI: 8.824-11.376) | <0.001 (F) | 0.813 (η^2^) |
| **MDA [ng/ug(*10^-3^)]** | 0.27 ± 0.05^b^  (95%CI: 0.2333-0.3203) | 0.24 ± 0.01^b^  (95%CI: 0.2350-0.2494) | 0.76 ± 0.51^a^  (95%CI: 0.3356-1.1854) | 1.08 ± 0.20^a^  (95%CI: 0.9158-1.2502) | 0.24 ± 0.03^b^  (95%CI: 0.2112-0.2692) | <0.001 (F) | 0.690 (η^2^) |
| **GR**  **[ng/ug(*10^-3^)]** | 3.36 ± 1.37^b^  (95%CI: 2.2106-4.5161) | 2.08 ± 0.66^b^  (95%CI: 1.5269-2.6348) | 12.77 ± 7.22^a^  (95%CI: 6.7336-18.800) | 12.45 ± 3.25^a^  (95%CI: 9.740-15.169) | 3.05 ± 1.01^b^  (95%CI: 2.2110-3.8985) | 0.001 (H) | 0.756 (ε²) |
| **NRF2 [ng/ug(*10^-3^)]** | 0.34 ± 0.04^b^  (95%CI: 0.2851-0.3834) | 0.21 ± 0.05^b^  (95%CI: 0.1722-0.2511) | 1.76 ± 1.19^a^  (95%CI: 0.7668-2.7482) | 1.57 ± 0.80^a^  (95%CI: 0.9021-2.2465) | 0.34 ± 0.04^b^  (95%CI: 0.3076-0.3749) | <0.001 (H) | 0.840 (ε²) |
| **KEAP1 [ng/ug(*10^-3^)]** | 124.91 ± 16.34^b^  (95%CI: 111.24-138.58) | 119.91 ± 19.47^b^  (95%CI: 103.63-136.19) | 446.97 ± 114.05^a^  (95%CI: 351.12-542.80) | 504.82 ± 62.08^a^  (95%CI: 452.82-556.62) | 125.28 ± 22.28^b^  (95%CI: 106.65-143.90) | <0.001 (F) | 0.690 (η^2^) |
| **Glucose (umol/ng)** | 507.50 ± 83.33^c^  (95%CI: 111.24-138.58) | 119.91 ± 19.47^c^  (95%CI: 103.63-136.19) | 446.97 ± 114.05^b^  (95%CI: 351.12-542.80) | 504.82 ± 62.08^a^  (95%CI: 452.82-556.62) | 125.28 ± 22.28^c^  (95%CI: 106.65-143.90) | <0.001 (F) | 0.942 (η^2^) |
| **Insulin (mIU/ng)** | 0.80 ± 0.10^b^  (95%CI: 0.7192-0.8952) | 0.65 ± 0.04^b^  (95%CI: 0.6132-0.6902) | 3.08 ± 0.92^a^  (95%CI: 2.2963-3.8494) | 3.19 ± 0.59^a^  (95%CI: 2.7042-3.6902) | 0.82 ± 0.35^b^  (95%CI: 0.5288-1.1244) | <0.001 (F) | 0.852 (η^2^) |

Total Antioxidant Capacity (TAC); Superoxide dismutase (SOD); Catalase (CAT); Glutathione peroxidase (GPx); Malondialdehyde (MDA); Glutathione reductase (GR); Nuclear factor erythroid 2-related factor 2 (NRF2); Kelch like ECH associated protein 1 (KEAP1); (1) NC, Negative Control group; (2) PC, Positive Control group; (3) PRO, Probiotic group; (4) CUR, Curcumin group; (5) PRO+CUR, Probiotic+Curcumin group; CI, Confidence interval; F, One-way ANOVA analysis; H, Kruskal-Wallis test; s.d., standard deviation. Data are shown as mean ± standard deviation (n = 8), whereas different letters signify substantially different values (*p*<0.05).

**Table S3.** The effects of probiotic and curcumin on pancreatic tissue biochemical parameters

| **Biochemical Parameters** | **^1^NC (n = 8)** | **^2^PC (n = 8)** | **^3^PRO (n = 8)** | **^4^CUR (n = 8)** | **^5^PRO+CUR (n = 8)** | **Test *p***  **value** | **Effect**  **Size** |
| --- | --- | --- | --- | --- | --- | --- | --- |
|  | **mean ± s.d. (CI)** | **mean ± s.d. (CI)** | **mean ± s.d. (CI)** | **mean ± s.d. (CI)** | **mean ± s.d. (CI)** |  |  |
| **TAC (U/ng)** | 0.19 ± 0.06^a^  (95%CI: 0.1383-0.2480) | 0.12 ± 0.05^ab^  (95%CI: 0.0772-0.1616) | 0.14 ± 0.05^ab^  (95%CI: 0.1053-0.1832) | 0.08 ± 0.06^b^  (95%CI: 0.0254-0.1384) | 0.16 ± 0.08^ab^  (95%CI: 0.0929-0.2303) | 0.017 (F) | 0.286 (η^2^) |
| **SOD [ng/ug(*10^-3^)]** | 0.33 ± 0.16^a^  (95%CI: 0.1944-0.4692) | 0.16 ± 0.04^a^  (95%CI: 0.1263-0.1983) | 0.29 ± 0.28^a^  (95%CI: 0.0460-0.5255) | 0.25 ± 0.12^a^  (95%CI: 0.1534-0.3530) | 0.27 ± 0.10^a^  (95%CI: 0.1847-0.3600) | 0.172 (H) | 0.068 (ε²) |
| **CAT [ng/ug(*10^-3^)]** | 10.05 ± 2.66^a^  (95%CI: 7.8297-12.2894) | 10.36 ± 4.25^a^  (95%CI: 6.8073-13.9094) | 10.76 ± 6.18^a^  (95%CI: 5.5919-15.9290) | 10.02 ± 4.93^a^  (95%CI: 5.8978-14.1374) | 9.36 ± 2.71^a^  (95%CI: 7.0946-11.4412) | 0.978 (F) | 0.012 (η^2^) |
| **GPx (Uu/ng)** | 28.73 ± 15.33^a^  (95%CI: 7.5299-9.3339) | 7.04 ± 3.15^b^  (95%CI: 4.4186-9.6651) | 18.21 ± 14.43^ab^  (95%CI: 6.1471-30.2702) | 9.11 ± 3.26^b^  (95%CI: 6.3838-11.8408) | 12.84 ± 9.05^b^  (95%CI: 5.2758-20.3684) | 0.001 (F) | 0.387 (η^2^) |
| **MDA [ng/ug(*10^-3^)]** | 0.41 ± 0.09^a^  (95%CI: 0.3413-0.4935) | 0.21 ± 0.06^a^  (95%CI: 0.1568-0.2533) | 0.35 ± 0.26^a^  (95%CI: 0.1083-0.5460) | 0.28 ± 0.11^a^  (95%CI: 0.1932-0.3742) | 0.33 ± 0.15^a^  (95%CI: 0.2037-0.4491) | 0.110 (H) | 0.195 (ε²) |
| **GR**  **[ng/ug(*10^-3^)]** | 2.92 ± 1.79^a^  (95%CI: 1.4200-4.4235) | 3.84 ± 1.43^a^  (95%CI: 2.6468-5.0298) | 4.77 ± 4.49^a^  (95%CI: 1.0282-8.5291) | 5.58 ± 2.07^a^  (95%CI: 3.8267-7.3352) | 3.71 ± 2.10^a^  (95%CI: 1.9560-5.4774) | 0.114 (H) | 0.099 (ε²) |
| **NRF2 [ng/ug(*10^-3^)]** | 0.58 ± 0.24^a^  (95%CI: 0.3764-0.7917) | 0.29 ± 0.10^a^  (95%CI: 0.2015-0.3744) | 0.53 ± 0.27^a^  (95%CI: 0.3020-0.7601) | 0.40 ± 0.24^a^  (95%CI: 0.2064-0.6047) | 0.56 ± 0.21^a^  (95%CI: 0.3763-0.7327) | 0.064 (F) | 0.219 (η^2^) |
| **KEAP1 [ng/ug(*10^-3^)]** | 136.93 ± 42.69^a^  (95%CI: 101.24-172.61) | 84.59 ± 22.80^a^  (95%CI: 65.54-103.65) | 189.80 ± 194.81^a^  (95%CI: 26.94-352.67) | 96.41 ± 69.12^a^  (95%CI: 38.70-154.11) | 124.73 ± 71.56^a^  (95%CI: 64.90-184.56) | 0.389 (H) | 0.037 (ε²) |
| **Glucose (umol/ng)** | 939.69 ± 473.11^a^  (95%CI: 111.24-138.58) | 417.70 ± 32.81^a^  (95%CI: 390.17-445.03) | 783.03 ± 703.47^a^  (95%CI: 194.78-1371.02) | 783.41 ± 431.28^a^  (95%CI: 422.69-1144.10) | 759.23 ± 272.06^a^  (95%CI: 531.78-986.68) | 0.060 (H) | 0.296 (ε²) |
| **Insulin (mIU/ng)** | 0.79 ± 0.25^a^  (95%CI: 0.5900-1.0094) | 0.20 ± 0.08^b^  (95%CI: 0.1369-0.2744) | 0.56 ± 0.66^ab^  (95%CI: 0.0056-1.1137) | 0.44 ± 0.15^ab^  (95%CI: 0.3133-0.5760) | 0.66 ± 0.31^ab^  (95%CI: 0.3872-0.9363) | 0.025 (F) | 0.266 (η^2^) |

Total Antioxidant Capacity (TAC); Superoxide dismutase (SOD); Catalase (CAT); Glutathione peroxidase (GPx); Malondialdehyde (MDA); Glutathione reductase (GR); Nuclear factor erythroid 2-related factor 2 (NRF2); Kelch like ECH associated protein 1 (KEAP1); (1) NC, Negative Control group; (2) PC, Positive Control group; (3) PRO, Probiotic group; (4) CUR, Curcumin group; (5) PRO+CUR, Probiotic+Curcumin group; CI, Confidence interval; F, One-way ANOVA analysis; H, Kruskal-Wallis test; s.d., standard deviation. Data are shown as mean ± standard deviation (n = 8), whereas different letters signify substantially different values (*p*<0.05).
